# Albumen—Translation

**Published:** 1856-08

**Authors:** 


### ALBUMEN.

TRANSLATED BY B. DOWLER, M. D.

From the Journal de Medicale de Bordeaux.

M. J. Jaennel, Profeesor of Mat. Med. in the Medical School of Bordeaux, who has written several articles reviewing the new work of M. Mailhe, pharmaceutist to the Emperor—(*Chimie appliquee a la physiologie et a la therapeutique*) quotes and adopts from this work the following conclusions concerning the behavior of albumen in the animal economy:

1. Normal Albumen or the serum of the blood and the white of the egg, do not traverse the animal membranes, *being insoluble.*

2. This state of insolubility ought to implicate the organism similarly with other substances which resist the law of endosmotic action, as fibrin, casein, and cruror among animals; starch and gluten in vegetables; substances known to have a globular organization, being held in suspension in liquids which serve as their vehicles.

3. As with these globular bodies, so albumen ought, in order to interpenetrate the economy, to undergo such modifications as will render it soluble and fit for the process of assimilation.

Thus normal albumen being insoluble and not amenable to endosmosis, is, when modified by a ferment (*pepsin*) not only rendered soluble but traverses the membranes perfectly.

4. By following out these transformations, albumen will be found in the animal economy in three conditions, having very distinct characteristics—namely, normal albumen, modified or *caseiforme* albumen, and albumenose.

Thus, says the reviewer, has M. Mailhe, thrown light upon the curious parallelism which exists between the vital processes of the two kinds of aliments, the amylaceous and the albuminous. The pepsin acid is like the distaste of albumen. The consequences resulting from these facts are of great physiological and pathological value.

M. Mailhe arranges medicinal substances into two classes, namely, the "*fluidifiants*" and the "*coagulants.*"

